# Neuromechanical Models of Mild Traumatic Brain Injury Conditioned on Reaction Time: A Systematic Review and Meta-Analysis

**DOI:** 10.3390/jcm13247648

**Published:** 2024-12-16

**Authors:** Avinash Baskaran, Ross D. Hoehn, Chad G. Rose

**Affiliations:** 1 Mechanical Engineering Department, Auburn University, Auburn, AL 36849, USA; azb0180@auburn.edu; 2 Pison Technology, Boston, MA 02111, USA; ross.hoehn@pison.com

**Keywords:** traumatic brain injury, neuromonitoring, rehabilitation

## Abstract

The accurate, repeatable, and cost-effective quantitative characterization of mild traumatic brain injuries (mTBIs) is crucial for safeguarding the long-term health and performance of high-risk groups, including athletes, emergency responders, and military personnel. However, gaps remain in optimizing mTBI assessment methods, especially regarding the integration of neuromechanical metrics such as reaction time (RT) in predictive models. **Background/Objectives:** This review synthesizes existing research on the use of neuromechanical probabilistic models as tools for assessing mTBI, with an emphasis on RT’s role in predictive diagnostics. **Methods:** We examined 57 published studies on recent sensing technologies such as advanced electromyographic (EMG) systems that contribute data for probabilistic neural imaging, and we also consider measurement models for real-time RT tracking as a diagnostic measure. **Results:** The analysis identifies three primary contributions: (1) a comprehensive survey of probabilistic approaches for mTBI characterization based on RT, (2) a technical examination of these probabilistic algorithms in terms of reliability and clinical utility, and (3) a detailed outline of experimental requirements for using RT-based metrics in psychomotor tasks to advance mTBI diagnostics. **Conclusions:** This review provides insights into implementing RT-based neuromechanical metrics within experimental frameworks for mTBI diagnosis, suggesting that such metrics may enhance the sensitivity and utility of assessment and rehabilitation protocols. Further validation studies are recommended to refine RT-based probabilistic models for mTBI applications.

## 1. Introduction

**Figure 1:**
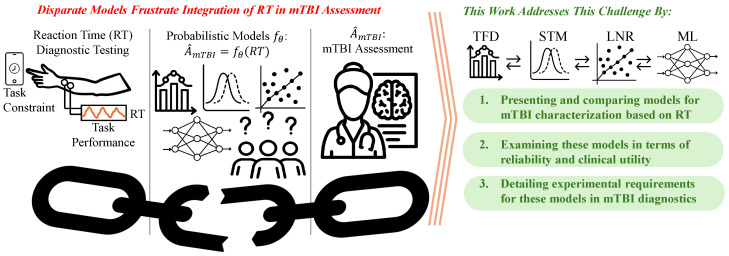
Existing mTBI assessment tools quantify patients’ reaction dynamics through physical observations by clinicians and are often overly coarse, generalizing, and challenging to perform accurately at the point of injury. Novel methods, including myography and portable neuro-imaging, have enabled robust, fine, and accurate measurement of reaction dynamics associated with mTBI at the point of injury, but the scientific literature reports a wide variety of disparate modeling approaches that frustrate methods to integrate RT into conventional mTBI assessment. This results in a broken chain between fast, reliable RT measurement and mTBI assessment. This work addresses this challenge by presenting and comparing models in the literature for mTBI characterization based on RT, examining the reliability and clinical utility of these models and detailing experimental requirements for using these models in mTBI diagnostics. This provides engineers, clinicians, and researchers with a guide to selecting and implementing mTBI models.

Mild traumatic brain injury (mTBI) occurs when an acute impact disrupts normal brain function. Short-term symptoms of mTBI can include headache, dizziness, and memory loss. Long-term symptoms can include post-concussion syndrome (PCS), which is characterized by persistent physical, cognitive, and emotional deficits. These symptoms can severely hinder the performance of daily tasks. The economic burden of mTBI includes long-term medical care and rehabilitation and lost productivity, which, together, can reach billions of dollars in the U.S. [1]. In addition, up to 50 million children and adolescents participate in contact sports [2], where mTBI accounts for 12% of sports-related injuries and 25% of all head injuries [3]. Many of these individuals never seek the assistance of medical professionals, chiefly as their head injury is undiagnosed and ignored. These silent concussions invite the individual to return to their standard activities—including the activity during which the concussion was experienced—possibly leading to additional subsequent head injuries and mTBI. This puts vulnerable populations, particularly youth, at greater risk [2]. This work presents and compares models in the literature for mTBI characterization based on reaction time to provide engineers, clinicians, and researchers with a guide to selecting and implementing mTBI models (Figure 1).

Some terminology used in the presentation and discussion of the results of this work is summarized in Table 1 to assist the reader.

### 1.1. Epidemiology of mTBI

The forces experienced by the brain during mTBI lead to mechanical stress on neural structures, including axons and blood vessels, causing diffuse axonal injury, which is characterized by axonal swelling and disconnection. This results in impaired neuronal communication and altered cerebral blood flow [4]. Damage to the blood–brain barrier can trigger harmful immune responses, disrupt neurotransmitter release, and impair metabolic functions, leading to cellular necrosis [4,5]. Further, mTBI also initiates a neurophysiological cascade, where injury to interconnected brain structures compounds over time, leading to long-lasting effects on cognitive and motor function [6].

### 1.2. Diagnostic Criteria and Assessment

Traditional diagnostic criteria used to assess mTBI are reliant on subjective assessments such as loss of consciousness (LOC) which can be highly intermittent, post-traumatic amnesia (PTA), and post-concussion syndrome (PCS). Gross grading scales, such as the Glasgow Coma Scale (GCS) and Sport Concussion Assessment Tool (SCAT), use arbitrary metrics like verbal responses, which may not accurately reflect mTBI severity and which may struggle to capture the long-term consequences of mTBI [7]. These subjective measures are prone to variability and may not offer sufficient granularity for personalized treatment. Recent advancements in sensorized, quantitative evaluation methods aim to address these limitations by incorporating objective measurements such as fMRI, CT, diffusion tensor imaging (DTI), electromyography (EMG), and motion capture [8]. These tools offer a more precise assessment of mTBI severity and allow for early detection and continuous monitoring of recovery.

### 1.3. Reaction Time (RT) as a Neuromechanical Measurand

Reaction time (RT) serves as a key neuromechanical measurand in assessing mTBI, as it quantifies the latency between a neuromotor stimulus and the corresponding motor response. RT encompasses sensory reception, neural transmission, central processing, and motor execution, reflecting the entire sensorimotor feedback loop. RT deficits are a well-established symptom of mTBI, where cognitive and motor impairments lead to prolonged responses. Diffuse axonal injury, neurotransmission disruptions, and imbalances in excitation–inhibition processes contribute to delayed RT in mTBI patients [9].

Given its involvement in both cognitive and motor pathways, RT serves as a sensitive marker for detecting subtle neuromechanical changes post-mTBI, where other measures may fail to capture cognitive–motor delays in response to external stimuli. Individuals suffering from mTBIs can exceed 250 ms in RT as recorded in simple reaction time (SRT) tests and other psychomotor vigilance tests (PVTs), compared with 180–200 ms for healthy individuals [10]. These detectable RT deviations can be exploited to track an individual’s recovery to a healthy state post-injury. RT tests are available in some concussion protocols but are often administered after the injury and not provided as a point-of-injury tool. Additionally, these tests are built into touchscreen devices which can be challenging to navigate for both clinicians and patients [11]. However, advancements in high-fidelity electromyographic sensing and motion capture technologies allow for precise measurement of RT, including premotor and motor latencies measured at the point of injury, providing a robust metric for tracking recovery. Wearable devices, such as the Pison READY [12], enable real-time, continuous monitoring of RT, opening new possibilities for rapid experimentation and rehabilitation interventions. As an integrative metric, RT can inform rehabilitation protocols, guide return-to-play decisions, and improve long-term mTBI management.

### 1.4. Objective and Organization of This Review

This review seeks to provide insights not covered in similar works [13,14,15,16], which focus on neuroimaging and other costly and challenging input spaces for mTBI prognostics, broadly scoped neuromuscular modeling, neurophysiological factors, and attentional dynamics reaching beyond RT. We focus on quantitative and qualitative characterization of neuromechanical models, with an emphasis on RT as a reliable, cost-effective alternative measurand to traditional neuroimaging measures. Specifically, we explore neuromotor changes before and after mTBI that directly impact RT, as RT metrics offer critical insights into mTBI’s impact on cognitive–motor coupling, which may be much more cost-effective to measure [17,18].

Achieving standardized mTBI characterization via sensor-based neuromuscular data requires an intermediary measurement model to capture the correlation between sensor measurements and mTBI characteristics [19]. In this work, we characterize such approaches through a comprehensive systematic review of the technical literature. To strengthen the utility of RT in concussion symptom tracking, this review highlights the significant body of literature dedicated to RT as a diagnostic measurand in mTBI assessment. RT directly reflects disruptions in neural pathways caused by concussion, providing a non-invasive, cost-effective measure of cognitive–motor deficits. Longitudinal studies demonstrate that RT delays persist through various stages of recovery, often paralleling or even preceding improvements detected by neuroimaging and biochemical markers. Wearable technologies, such as high-fidelity EMG and motion capture systems, now offer real-time RT monitoring in both clinical and athletic settings, allowing for the continuous assessment of concussion symptoms without the need for costly equipment or invasive tests. RT has proven effective not only in diagnosing mTBI but also in monitoring recovery, guiding return-to-play decisions, and customizing rehabilitation protocols by linking improvements in RT with functional recovery milestones. Recent advancements in predictive modeling using RT metrics, particularly probabilistic frameworks and machine learning approaches, have further enhanced the ability to assess concussion severity and forecast recovery trajectories. Integrating RT into broader concussion and mTBI management strategies can provide a robust and scalable solution, offering both diagnostic sensitivity and practical applicability in real-world settings.

To address the complexities of RT-related predictive models for mTBI prognosis, it is essential to recognize the steps involved in model development, validation, and application across diverse populations. Predictive models are built using training datasets, often derived from sensor data capturing neuromechanical metrics like reaction time (RT), and undergo feature selection to identify key predictors of mTBI outcomes. Once built, these models are typically validated through cross-validation techniques, such as k-fold cross-validation or leave-one-out methods, to assess generalization performance across different samples. For clinical applicability, external validation using independent datasets is necessary to test model robustness in new populations. Additionally, metrics like accuracy, sensitivity, specificity, and area under the ROC curve (AUC) provide quantitative assessments of model performance, ensuring that models are not only statistically significant but clinically relevant. It is also critical to adjust for potential confounders (e.g., age, gender, and sport type) and address imbalances in datasets to prevent bias. This rigorous approach to model development and validation ensures that RT-based models for mTBI prognosis are adaptable and effective across a range of real-world applications, increasing their utility in personalized care.

## 2. Methods

**Figure 2:**
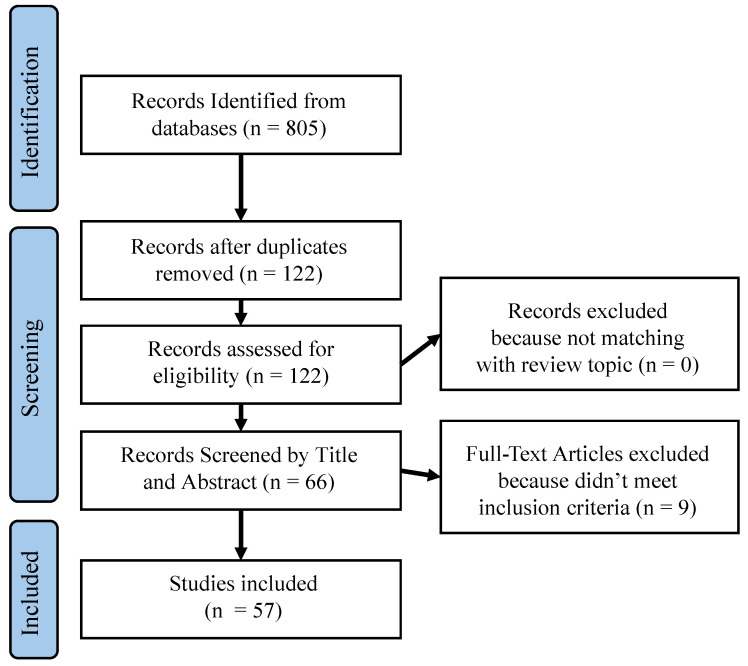
Preferred Reporting Items for Systematic Reviews and Meta-Analyses (PRISMA) flowchart illustrating the data retrieval protocol used in this work.

This systematic review and meta-analysis was conducted in accordance with the 2020 Preferred Reporting Items for Systematic Reviews and Meta-Analyses (PRISMA) guidelines. All applicable elements of the PRISMA checklist have been followed, and a completed PRISMA flow diagram is included in the flow diagram (Figure 2), which details the study selection process, including the number of studies screened, assessed for eligibility, and included in the final analysis. No registration was required for this review.

### 2.1. Search Strategy and Selection Criteria

We conducted a thorough search of several electronic databases to capture a wide range of studies pertaining to the objective quantitative characterization of mTBI. The databases included in our survey for their extensive coverage of studies on sports medicine, neurology, and biomedical engineering are PubMed and Scopus.

To ensure comprehensiveness and focus on sensor-based mTBI modeling, we used keyword and medical subject heading (MeSH) terms to capture studies incorporating terms related to “mTBI”, “diagnostic criteria”, “probabilistic models”, “reaction dynamics”, “neuroimaging”, “MRI”,“CT Scan”, “electromyography”, and “motion capture”. Specific search strings were adapted for each database to align with its indexing system and search capabilities. An example search string used in PubMed is

(“mild traumatic brain injury” OR “mTBI”) AND (“model”) AND (“reaction” OR “reaction time” OR “response”) AND (“neuroimaging” OR “MRI” OR “Magnetic Resonance Imaging” OR “CT Scan” OR “Computed Tomography” OR “electromyography” OR “EMG” OR “motion capture” OR “biomechanical analysis” OR “sensor”)

#### 2.1.1. Inclusion and Exclusion Criteria

Studies were selected based on the following inclusion criteria to ensure relevance: Published in peer-reviewed journals within the last 10 years, and specifically addressing the model-based assessment of mTBI with empirical results. Studies were excluded based on the following criteria: availability in English, not focused on mTBI, opinion pieces, editorials, and commentaries that did not provide empirical data or systematic reviews. The Preferred Reporting Items for Systematic Reviews and Meta-Analyses (PRISMA) flowchart that illustrates the data retrieval protocol is shown in Figure 2.

#### 2.1.2. Study Selection, Appraisal, and Data Extraction

First, titles and abstracts of collected articles were screened to remove duplicates and assess relevance, and then the remaining articles were subjected to full-text review to ensure suitability for inclusion according to the defined criteria. From each selected study, the study design, participant demographics, mTBI assessment model, and results were obtained and tabulated for comparison. A narrative synthesis was employed to integrate findings. To identify the strength and reliability of the evidence presented in the selected studies, randomized control trials were assessed using the Cochrane Risk of Bias tool Version 2 [20], and observational studies were evaluated through the 2014 Newcastle–Ottawa Scale [21]. This rigorous search strategy and selection criteria ensured a review of high-quality, relevant, and recent literature.

## 3. Results

### 3.1. Surveyed Studies

We conducted a comprehensive search, identifying 229 studies from PubMed and 576 studies from Scopus, aimed at exploring sensor-based modeling for mild traumatic brain injury (mTBI). After removing duplicates and screening abstracts and titles, 122 studies were deemed relevant. Full-text review led to the inclusion of 57 studies for detailed information extraction (Figure 2). All selected studies were published in peer-reviewed journals after 2015. The risk of bias (RoB) analysis and the Newcastle–Ottawa (N-O) scale evaluation were applied to to assess study quality. The RoB assessment was categorized into D1 (randomization), D2 (deviation from intended intervention), D3 (missing outcome data), and D4 (measurement of outcome), and the N-O scale included S (appropriateness of subject selection), R (randomization in study design), E (clarity of the exposure or intervention definition), C (control for confounding variables), A (reliability of outcome measurements), and F (adequacy of participant follow-up). Each metric was rigorously evaluated, and the results of this evaluation are listed in Table 2, with gray cells indicating medium to high risk of bias and white cells indicating low risk.

**Figure 3:**
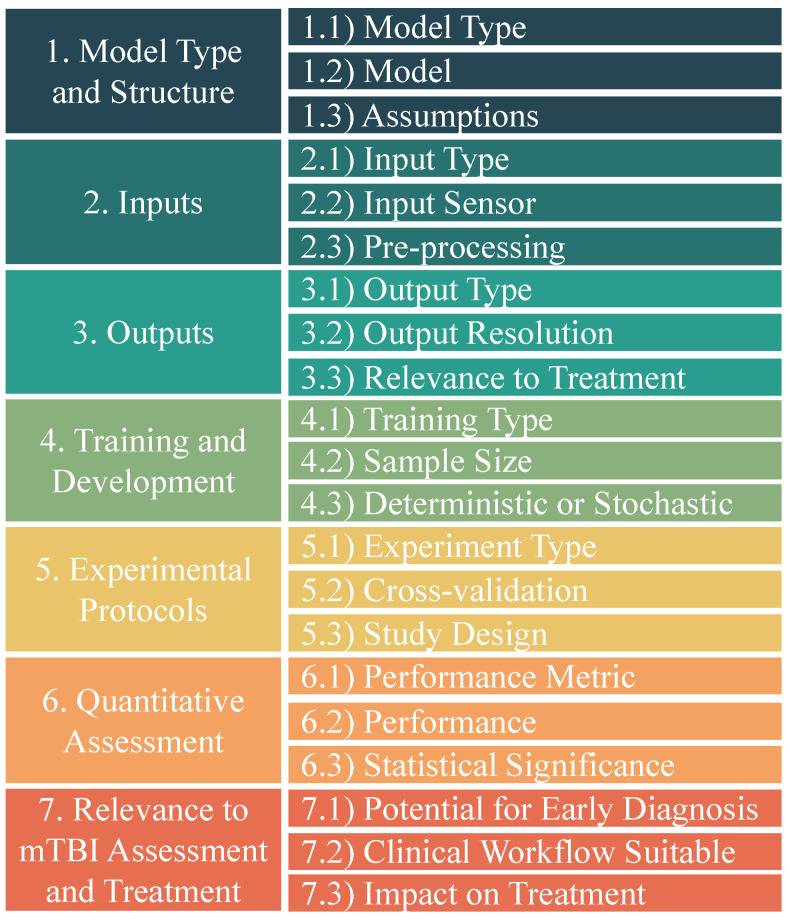
We extracted features pertaining to seven data categories (listed above) from each of the 57 studies selected for review. Quantitative items, including sample size, and statistical significance are used later on to characterize the neuromechanical models used in the studies.

Some studies, such as Cui et al. [23] and Dean et al. [25], exhibited elevated bias risk due to their reliance on simulation data, which lacked real-world validation and did not directly account for the complexities of human physiology. These studies also struggled controlling for external variables, which reduced the applicability of their findings to clinical scenarios. In general, many studies exhibited significant weaknesses in the RoB domains D2, D3, and D4. Specifically, studies tended to fail in D2 (deviations from intended interventions) due to inconsistent application of the intervention across study subjects, often resulting from protocol deviations or unintentional variations in exposure. In D3 (missing outcome data), studies often lacked rigorous follow-up, leading to incomplete datasets that undermined the integrity of the results—particularly when dropout rates were not properly addressed or accounted for in the analysis. Lastly, studies struggled in D4 (measurement of outcome) when the outcome variables were inadequately defined or measured with insufficient accuracy, such as using surrogate markers instead of direct clinical outcomes or relying on self-reported data without objective validation methods. These specific issues collectively contributed to a higher risk of bias across multiple studies. The N-O results showed that most studies performed well in E and A. The majority of studies used reliable methods to ascertain exposure and accurately assess outcomes. However, in terms of F some studies had insufficient follow-up duration or completeness, which could have impacted the robustness of their findings. Additionally, comparability was a weaker domain, as several studies failed to adequately control for confounding variables. A numerical model of mTBI severity was the most frequently studied outcome. Studies focused on mTBI severity at different time points during follow-up. The studies analyzed employed a range of models, inputs, and pre-processing techniques aimed at understanding the characterization of mTBI through sensor-based and neural data. We extracted features pertaining to seven data categories listed in Figure 3.

### 3.2. Model Types and Structures

First, each selected study was categorized with regards to an input space (a space of variables comprising the inputs to neuromechanical models), a model space (constituting types of models), and an output space (a space of variables comprising the outputs from neuromechanical models). The input space of the studies included sensorized movement-based functional assessments with stimuli-based task constraints (SBC) [22,25,26,28,29,30,31,32,33,34,36,37,38,39,58,64], reaction time task constraints (RTC) [19,23,27,35,41,42,43,44,45,46,48,50,52,53,55,57], and general cognitive task constraints (GCC) [24,40,47,49,51,54,56,61,62,63,65,66,67,68,69,71]. The model space includes machine learning (ML) [22,23,39], linear regression (LNR) [19,24,25,26,29,32,37,45,66,68,72,73,75], stochastic/ANOVA (STM) [27,28,30,31,34,35,38,40,42,44,46,47,49,50,51,52,53,54,55,57,58], and time frequency distribution (TFD) models [33,36,41,43,48,56,70,76]. The output space included performance characteristics of mTBI and mTBI severity such as cognitive performance metrics (CPM) [23,27,31,34,40,43,47,48,49,50,51,52], neural activity metrics (NAM) [22,24,28,30,35,37,41,45,53,54,55,56,57,58], behavioral and symptom metrics (BSM) [19,25,29,33,36,42,59,60,61,62,63,64], neuromotor and reaction metrics (NRM) [26,32,38,39,44,46,65,66,67,68,69,70], and imaging-based metrics (IBM) [24,26,35,39,71,72,73,74,75,76].

The input space represents the spectrum of tasks relevant to mTBI assessments particularly suited to eliciting measurable neuromechanical responses, allowing for a nuanced understanding of mTBIs’ effects. The model space reflects the need to handle complex, high-dimensional data from various modalities, such as neuroimaging and EMG. These models typically assume underlying relationships between inputs and outputs, which are either linear or stochastic, and allow for flexibility in interpreting the effects of injury across different domains. ML models, particularly, provide the potential for capturing non-linear relationships and discovering patterns that are not easily modeled by traditional methods. The output space represents the varied and multifaceted effects of mTBI. These outputs were selected for their capacity to reflect both cognitive and motor deficits, as well as structural and functional changes in the brain. The assumptions behind these choices likely included expectations that these metrics would be sensitive to the impacts of mTBI, providing reliable indicators of injury severity and recovery trajectories across different patient populations.

### 3.3. Data Preprocessing

The preprocessing of both the input and output data spaces is critical in ensuring the robustness of neuromechanical probabilistic models for mTBI assessment. Across the studies reviewed, various preprocessing steps were employed to handle the diverse nature of input and output signals, including normalization, feature selection, and signal filtering.

#### 3.3.1. Input Space Preprocessing

For neuroimaging and neuromechanical signals, such as those obtained through fMRI, EEG, and EMG, noise reduction was a key preprocessing step [31,36,47]. High-pass and low-pass filters were commonly used to remove low-frequency drift and high-frequency noise, respectively. Motion artifacts in fMRI data were corrected using realignment algorithms [30]. EMG signals were band-pass-filtered in the 20–450 Hz range to retain relevant motor control information while reducing noise from electrical interference [24,43].

Normalization was employed to scale the features of the input data, particularly in cases where variables had different units or magnitudes. For example, neuroimaging features such as fractional anisotropy (FA) values were normalized to a [0, 1] range to facilitate comparison across subjects and imaging sessions [26,28]. Similarly, reaction time (RT) metrics were normalized across trials to adjust for individual differences in baseline response times [33,39,49].

Feature extraction was applied to reduce the dimensionality of the input data, often utilizing techniques like principal component analysis (PCA) or independent component analysis (ICA) for neuroimaging data [19,41]. In studies that relied on EMG and motion capture, time-domain and frequency-domain features, such as mean absolute value and median frequency, were extracted from the raw signal to better characterize neuromuscular activity [35,46]. Studies employing machine learning models often utilized feature selection techniques such as recursive feature elimination (RFE) to identify the most relevant input variables for predicting mTBI outcomes [32,37].

#### 3.3.2. Output Space Pre-Processing

For studies that focused on classification tasks, continuous output measures such as reaction time (RT) or neuroimaging-derived metrics were often binarized into “mTBI”; and “non-mTBI” categories using established clinical thresholds [25,44,50]. Binarization facilitated the use of logistic regression models and other binary classifiers. Additionally, studies that aimed to predict symptom severity categorized outcomes into mild, moderate, and severe categories based on standardized scales [40,42].

To address imbalances in the dataset, particularly in the classification of mTBI severity, resampling techniques were employed. Oversampling techniques such as SMOTE (Synthetic Minority Over-sampling Technique) were used to ensure that minority classes (e.g., severe mTBI) were sufficiently represented during model training [27,48]. In some studies, bootstrapping methods were applied to enhance the robustness of the statistical models and provide more stable performance estimates [34,38].

K-fold cross-validation, was utilized to assess the generalizability of predictive models across the majority of studies. This technique involved partitioning the dataset into k subsets, training the model on k-1 subsets, and validating it on the remaining subset. This process was repeated k times, ensuring that each data point was used for both training and validation. Common choices for the number of folds ranged from 5 to 10, depending on the size of the dataset and the complexity of the model [23,29,45]. Studies also reported utilizing leave-one-out cross-validation for small sample sizes [51,53].

In studies dealing with limited datasets, data augmentation techniques were employed to artificially expand the training set. Techniques included jittering and time-warping for time-series data such as EMG and reaction time signals and spatial transformations for neuroimaging data. This increased the diversity of the training data, helping the models generalize better to unseen data [39,52].

Missing data were addressed through imputation methods in several studies. Simple imputation techniques, such as mean or median imputation, were commonly used for missing input values [30,47]. For more complex datasets, such as those involving longitudinal neuroimaging or repeated reaction time measures, more sophisticated approaches like multiple imputation were utilized to avoid biasing the results [43,49].

### 3.4. Experimental Framework

Next, we highlight the rigorous experimental frameworks employed to explore the neuromechanical underpinnings of mild traumatic brain injury (mTBI). Many studies utilize reaction time (RT) as the primary behavioral metric for mTBI characterization. For instance, standardized RT tasks, such as simple stimulus–response pairs or complex decision-making tasks, have been widely adopted due to their sensitivity to cognitive–motor impairments post-injury [38]. Variations in RT are attributed to factors such as task complexity, the equipment used, and participant demographics. Studies have shown direct correlations between increased task complexity and greater RT variability [25], with some tasks reporting standard deviations exceeding 100 ms [25,37]. These findings underscore the importance of carefully designed experimental tasks that isolate specific cognitive processes while minimizing confounding variables such as fatigue or attentional demands [78].

In addition to RT, assessments of postural control have been incorporated to elucidate the effects of mTBI on motor function. Postural assessments, like the somatosensory organization test, have been shown to reveal deficits in visual, somatosensory, and vestibular inputs post-injury [30]. Imaging methods, such as electroencephalography (EEG), magnetoencephalography (MEG), and functional magnetic resonance imaging (fMRI), have played pivotal roles in capturing neural activity patterns in mTBI patients. Resting-state EEG and MEG recordings, for example, reveal disrupted connectivity in networks critical for working memory and attention. In particular, findings suggest diminished beta band spectral content and reduced temporal coordination of burst states in mTBI patients during motor tasks [22,38]. These sophisticated techniques have provided crucial insights into network-level disruptions post-injury, offering a more nuanced understanding of neural deficits not visible through structural imaging alone [22,27].

Computational network models have also emerged as powerful tools for understanding the mechanisms linking mTBI to observed neurobehavioral deficits. For instance, large-scale biologically plausible neuronal network models incorporating intra- and interhemispheric organization, such as callosal axons, have been used to simulate the impact of axonal injury on cognitive function. These models reveal that mTBI can impair interhemispheric information exchange, particularly in the corpus callosum, which correlates with increased RTs and diminished cognitive performance [23]. Such models not only complement experimental neurophysiological data but also provide insights into how structural disruptions translate to functional deficits.

Animal models, particularly rodent models, have been integral to advancing our understanding of mTBI. These models replicate key features of mTBI in humans, such as loss of consciousness and amnesia, allowing for controlled studies of long-term cognitive effects [19,26]. Researchers have adapted methodologies like vibrotactile stimulus-evoked recordings to measure neural activity in response to tactile stimuli, drawing parallels between animal and human studies in terms of cognitive and motor responses to injury [19]. The experimental frameworks adopted in mTBI research span a wide range of behavioral, neurophysiological, and computational methodologies, contributing unique insights into the cognitive and motor dysfunction following mTBI. These robust frameworks facilitate a comprehensive understanding of mTBI and support diagnostic and therapeutic interventions for mitigating its long-term effects.

**Figure 4:**
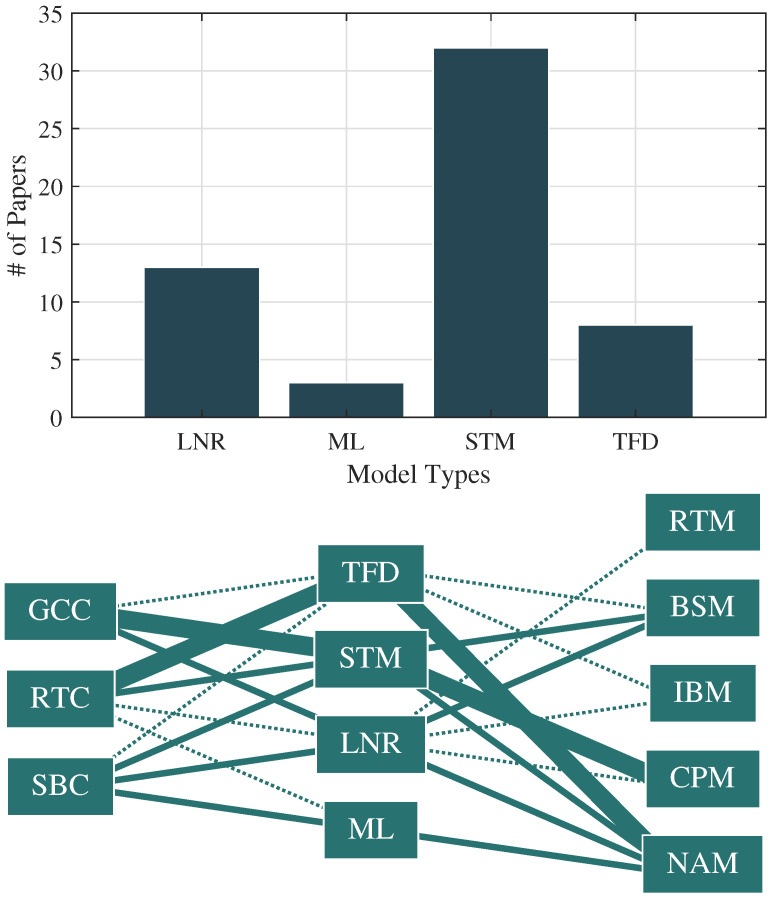
Above, a histogram of model types used in the selected studies (**top**) and a map linking inputs to outputs through models used in the selected studies (**bottom**) are shown. The lines shown in the map represent hypotheses. The thicker dark lines represent the most common hypotheses evaluated, the thinner dark lines represent the lesS common, and the dotted lines represent the least common hypotheses evaluated. The most commonly evaluated hypotheses were that experimental conditions including cognitive constraints (GCC, e.g., distraction tasks) and reaction time constraints (RTC, e.g., timed tasks) can be linked to neural activity (NAM) and cognitive performance (CPM) through time–frequency analysis and stochastic modeling.

In Figure 4, we provide a histogram of the model types used in the selected studies (top), and a topographical map linking the inputs and outputs used through their respective models in the selected studies (bottom) is shown. The lines in the map represent hypotheses evaluated in the selected studies (e.g., STM can be used to model CPM from GCC). The thicker dark lines represent the most common hypotheses evaluated, and the thinner lines represent the least common hypotheses evaluated. The most commonly evaluated hypotheses were that cognitive and reaction time constraints can be linked to neural activity and cognitive performance through time–frequency analysis and stochastic modeling. This reflects the poorer performance of many ML models in high-dimensional data spaces such as biometrics, and the high statistical correlation between mTBI symptoms and neuromechanical measurands.

### 3.5. Meta-Analysis

To rigorously evaluate the performance of various models and hypotheses within the dataset, we applied a comprehensive meta-analytical approach. This involved employing advanced statistical techniques to ensure robust assessments, particularly when hypotheses overlapped and sample sizes varied. Given the repetitive testing of similar hypotheses across different models, we grouped data based on input–model–output combinations. For each group, we combined *p*-values using both Fisher’s Method and Stouffer’s Z-Score. Fisher’s Method aggregates *p*-values by summing their logarithmic transformations, yielding a statistic that follows a $\chi^{2}$ distribution with 2k degrees of freedom, where *k* is the number of models in the group:

(1) $$\chi^{2}=-2\sum_{i=1}^{k}\ln\left(p_{i}\right)$$

Stouffer’s Z-Score, on the other hand, accounts for differences in sample sizes by applying weighted significance tests. This method combines *p*-values by weighting them according to sample sizes:

(2) $$Z=\frac{\sum w_{i}Z_{i}}{\sqrt{\sum w_{i}^{2}}}$$

where $Z_{i}$ is the Z-score corresponding to the *i*-th *p*-value, and $w_{i}=\sqrt{n_{i}}$ is the weight based on sample size $n_{i}$. This provides a weighted cumulative significance measure that adjusts for sample size variability.

Additionally, we employed Bayesian Model Averaging (BMA) to address uncertainty across models and hypotheses. BMA computes a weighted average of posterior probabilities across all models, allowing for a probabilistic interpretation of results. For each input–model–output combination, we calculated the likelihood of the hypothesis being true, incorporating *p*-values and sample sizes into the posterior distribution.

To handle the hierarchical nature of the data, where the same hypotheses were tested across multiple models and conditions, we used a mixed-effects modeling approach. This separated fixed effects (such as the influence of specific inputs, models, and outputs) from random effects, which accounted for variability across models:

(3) $$Y_{ijk}=\beta_{0}+\beta_{1}X_{\mathrm{input}}+\beta_{2}X_{\mathrm{model}}+\beta_{3}X_{\mathrm{output}}+u_{ij}+\epsilon_{ijk}$$

where $Y_{ijk}$ represents the response variable; $\beta_{0}$ represents the fixed intercept; $\beta_{1}$, $\beta_{2}$, $\beta_{3}$ represent the fixed effects for input, model, and output; and $u_{ij}$ represents the random effect for each input–model–output combination, with $\epsilon_{ijk}$ as the error term.

Finally, we calculated the False Positive Risk (FPR) for each input–model–output combination. This adjusted the significance level by considering the prior probability of the hypothesis being true, the observed *p*-values, and sample sizes:

(4) $$FPR=\frac{1}{1+\left(\frac{1-\pi }{\pi }\times \frac{1-p}{p}\times \frac{n}{n_{\mathrm{ref}}}\right)}$$

where π is the prior probability of the hypothesis being true, *p* is the observed *p*-value, *n* is the sample size, and $n_{\mathrm{ref}}$ is the reference sample size. This calculation provides an estimate of the probability that a significant result is a false positive. The results of the meta-analysis, as summarized in Table 3, reveal several key insights into the performance of different model groups when evaluating various input–model–output combinations.

The STM model group consistently demonstrates strong performance, particularly with CPM and NAM outputs, as reflected by Fisher *p*-values as low as 0.025 and significant Stouffer Z-scores. Notably, STM1 and STM3 highlight significant results with CPM outputs, indicating that the combination of SBC and RTC inputs tends to yield robust performance across multiple measures. The LNR model group, on the other hand, shows solid associations with NEU and BSM outputs, with LNR1 achieving a Fisher *p*-value of 0.028 and a high Stouffer Z-score of 2.54, suggesting that the RTC and SBC inputs in LNR models contribute to strong neurological output significance. In contrast, the ML models demonstrate more moderate performance, particularly with BEH outputs, with *p*-values around 0.033, indicating a weaker but still notable correlation between the inputs and outputs. The TFD model group stands out with strong results for NEU and IBM outputs, particularly in TFD1, which has the lowest *p*-value in the entire analysis (0.020) and a robust BMA weight of 0.70, suggesting a high level of confidence in its predictions. Across all model groups, Bayesian Model Averaging (BMA) weights consistently demonstrate that models achieving lower *p*-values also carry higher BMA weights, underscoring the probabilistic reliability of these models. The False Positive Risk (FPR) values remain low across the board, suggesting that the risk of false positives is minimal, thereby supporting the validity of the findings.

The ML models, while still performing moderately well, appear to struggle slightly in capturing the full complexity of the outputs, as evidenced by somewhat higher *p*-values and lower BMA weights compared to STM and LNR models. This suggests that while machine learning can be powerful, it may require larger sample sizes or more refined input features to achieve the same level of statistical significance in these contexts. Finally, the TFD models shine in their ability to analyze complex time-varying signals, as evidenced by their strong performance in predicting NEU and IBM outputs. The lowest Fisher *p*-value (0.020) observed in TFD1 highlights the model’s ability to effectively handle frequency-based data, making it particularly well suited for applications where time–frequency relationships are important. Across all model types, the strong BMA weights and low False Positive Risk values underscore the robustness of the findings, suggesting that each model type has its own strengths depending on the nature of the inputs and outputs it is tasked with analyzing.

**Figure 5:**
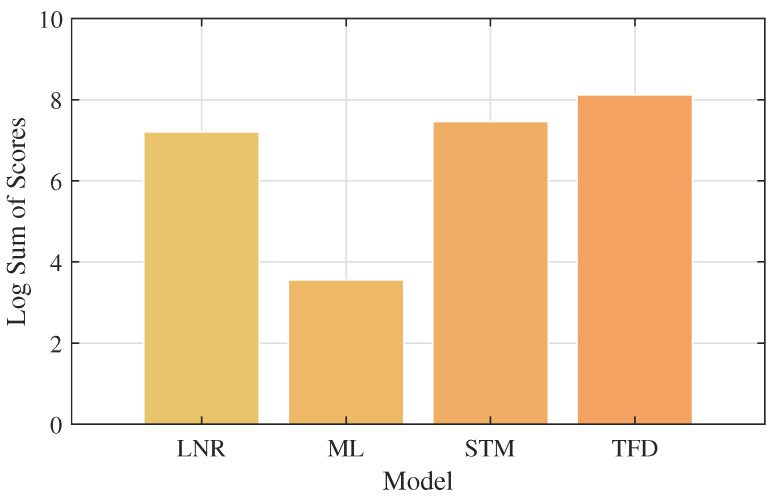
Visual representations of the log sum of Stouffer Z-scores and BMA weights across the various models tested are shown. The study indicates high utility and reliability of analytical time–frequency models and less reliability of machine learning models.

Figure 5 represents the distribution and performance of different model types (e.g., ML, LNR, STM, TFD) based on their *p*-values and sample sizes across 39 studies. The horizontal axis represents the models, while the vertical axis represents the logarithmic sum of scores for each model type, combining their *p*-values and sample sizes. The height of the bars in the plot reflects the overall significance of each model type: higher bars indicate a larger combined contribution from both lower *p*-values and larger sample sizes.

## 4. Critical Assessment of Gaps, Limitations, and Inconsistencies in mTBI Research

Despite advancements in understanding mild traumatic brain injury (mTBI), gaps and limitations result in inconsistencies in the literature. One significant area of contention is the diverse range of methodologies used to assess mTBI, particularly regarding neural dynamics and cognitive outcomes. Studies often employ differing neuroimaging techniques such as MEG, fMRI, and EEG, leading to variations in the reported biomarkers and mechanisms underlying mTBI. For instance, while MEG studies highlight disrupted beta bursts and altered burst connectivity as critical markers of mTBI [22], other studies using computational network models focus on the slowing of network rhythms and increased theta-to-alpha power ratios as primary indicators [23]. These discrepancies raise concerns about the consistency and generalizability of mTBI biomarkers.

A major limitation to robust modeling is variability in mTBI symptoms and outcomes, which is exacerbated by variations in injury mechanisms, severity, and recovery timelines. While some studies demonstrate persistent cognitive impairments such as reduced working memory performance and attention deficits up to several years post-injury [35], others show near-complete recovery in cognitive functions within weeks [24]. This variability in findings reflects the need for more standardized and longitudinal assessments that account for individual differences in injury mechanisms, such as blast versus blunt force trauma [34].

Additionally, the reliance on self-reported symptoms and baseline testing introduces biases and inconsistencies. For example, some studies demonstrate that baseline testing can yield misleading results due to practice effects, especially when repeated multiple times [46]. Conversely, studies relying on normative data for concussion assessments suggest that individualized baseline testing may not always be necessary for accurate diagnosis, as normative data can serve as a reliable alternative in some cases [41]. However, the reliance on normative data is criticized for lacking sensitivity to subtle neurophysiological changes, particularly in high-performance athletes, where cumulative subconcussive impacts might go undetected until symptoms manifest [43].

A gap in current methods is the quantification of long-term repeated mild TBIs, particularly in populations such as military personnel and athletes where there is an elevated risk due to chronic cognitive dysfunctions and executive function impairments, as well as an increased risk of developing neurodegenerative conditions [34]. Animal models of mTBI attempt to replicate these conditions but often fail to capture the complexity of human injuries and the long-term cognitive effects [19].

Finally, the integration of advanced machine learning models and computational approaches in mTBI research remains in its infancy. Although machine learning has shown promise in predicting mTBI outcomes, especially in complex, multimodal datasets [26], many studies fail to account for the inherent variability in input signals such as neuroimaging data, cognitive task performance, and motor coordination metrics. This limits the accuracy and generalizability of predictive models across diverse patient populations.

These limitations underscore the need for more standardized protocols, longitudinal studies, and advanced modeling techniques to address the multifaceted nature of mTBI and improve diagnostic accuracy, treatment, and prognostic outcomes.

## 5. Discussion

### 5.1. Neuromechanical Probabilistic Models

This section explores the six key neuromechanical probabilistic models analyzed in this review: stochastic/ANOVA models (STMs), machine learning (ML) models, linear regression (LNR), and time–frequency decomposition (TFD) models. Each model’s contribution to mTBI characterization, computational requirements, and ability to manage complex neuromechanical data is assessed. Emphasis is placed on the suitability of these models for large-scale data inference and real-time applications.

#### 5.1.1. Stochastic Models (STM)

Stochastic models capture the inherent randomness and uncertainty of mTBI data, particularly noisy neuromechanical signals like electromyography (EMG) and magnetoencephalography (MEG). These models are essential for real-time signal processing, offering flexibility in modeling noisy data, though they demand higher computational power, particularly in large-scale studies using MEG and fMRI. Their complexity scales as O(n·*p*·k), where *n* is the number of observations, *p* is the stochastic parameters, and *k* is the iterations required for convergence. Despite their computational intensity, they remain highly effective for tasks requiring real-time analysis and high variability modeling [22].

#### 5.1.2. Machine Learning Models (ML)

Machine learning models, particularly neural networks and decision trees, offer powerful non-linear modeling capabilities. These models are essential for uncovering hidden patterns in mTBI data, particularly where interactions between motor control and cognitive function are non-linear. Machine learning models are scalable with time complexity O(L·*n*·p), where *L* is the number of layers, *n* is the number of samples, and *p* is the input features. However, these models demand significant memory resources, especially when handling large datasets like EMG or diffusion tensor imaging (DTI). Their ability to model complex interactions makes them highly suitable for predictive analytics in mTBI prognosis [26].

#### 5.1.3. Linear Regression (LNR)

Linear regression assumes a linear relationship between dependent and independent variables. Despite its simplicity, it remains computationally intensive for large-scale neuromechanical data, with a time complexity of $O(n^{3})$. This model is not ideal for mTBI, where non-linear effects and interactions dominate. As a result, linear regression must often be augmented with interaction terms or replaced by more flexible models like GLMs to achieve better performance in predicting mTBI outcomes [27].

#### 5.1.4. TFD Models (TFD)

Time–frequency decomposition (TFD) models are widely used for analyzing transient neuromechanical signals, particularly in EEG or EMG data. These models are efficient, with a typical complexity of O(nlogn), and enable the characterization of dynamic states across time scales. This makes them suitable for mTBI research, where capturing oscillatory neural dynamics and motor responses is key. However, the choice of technique, such as wavelet or Fourier decomposition, can significantly impact accuracy and computational efficiency [25].

#### 5.1.5. ANOVA (ANV)

ANOVA is used to compare group means, making it a common tool in experimental neuroscience. With the time complexity of O(n), ANOVA models are computationally efficient but are less suitable for complex neuromechanical data that often violate assumptions like homogeneity of variance. Advanced variants, such as mixed-effects models, offer some flexibility but require careful handling to ensure assumptions hold [23]. A more complete description of time complexity, memory usage, time per data point, and relative scalability of each of the considered models is provided in Table 4.

### 5.2. Signal Characteristics of Inputs and Outputs

This section provides detailed descriptions of the key signal characteristics of inputs: neuroimaging (NEU), motor coordination (MOT), and cognitive tasks (COGs)—and outputs—cognitive performance metrics (CPMs), neural activity metrics (NAMs), and motor task performance (MTP). Understanding these characteristics is vital for developing effective neuromechanical probabilistic models for mTBI.

#### 5.2.1. Neuroimaging (NEU)

Neuroimaging data consist of MRI metrics, diffusion tensor imaging (DTI), and functional connectivity measures. These typically have high spatial resolution, with voxel sizes ranging from 1 to 2 mm in structural MRI and temporal resolutions of 1–2 seconds for functional MRI. They exhibit wide dynamic ranges, with fractional anisotropy (FA) values between 0 and 1. Preprocessing steps like motion correction and smoothing improve the signal-to-noise ratio (SNR).

#### 5.2.2. Motor Coordination (MOT)

Motor coordination data are derived from kinematic tracking and EMG signals, with typical sampling rates between 100 Hz to 1000 Hz to capture fine movement details. Accelerometer signals in these tasks range from −16 g to +16 g. EMG signals, band-pass filtered to remove electrical noise, are essential for studying motor performance, particularly in neuromechanical contexts.

#### 5.2.3. Cognitive Tasks (COGs)

Cognitive tasks generate performance data tied to memory, attention, and decision-making. These signals typically involve metrics like response accuracy, reaction times, and error rates during tasks. Temporal precision in these tasks is key, with millisecond accuracy needed for cognitive performance analysis.

#### 5.2.4. Cognitive Performance Metrics (CPMs)

Cognitive performance metrics quantify execution in cognitive tasks, such as response accuracy, reaction times, and error rates. These are highly sensitive to task design and can vary based on factors like attention span, fatigue, or cognitive load.

#### 5.2.5. Neural Activity Metrics (NAMs)

Neural activity metrics involve tracking brain activity using techniques like EEG, fMRI, or MEG. EEG offers sub-millisecond temporal precision, while fMRI captures slower hemodynamic changes, providing a comprehensive view of neural dynamics in response to tasks.

#### 5.2.6. Motor Task Performance (MTP)

Motor task performance metrics assess movement execution, precision, and timing. These typically involve kinematic properties like amplitude, velocity, and acceleration. High temporal resolution is crucial for accurately capturing fast-paced motor actions, with sub-millisecond timing necessary for certain fine motor tasks. A more complete description of the input and output signals assessed in this work, including the key characteristics, amplitude, and resolution of each signal type is provided in Table 5.

## 6. Practical Implications

The findings of this systematic review can enhance clinical practice, sports safety protocols, and rehabilitation for mTBI. By synthesizing the latest advances in neuromechanical probabilistic models, this review highlights key areas for refinement and implementation in emerging protocols to improve diagnosis, treatment, and prevention strategies. One of the primary implications for clinical practice is the integration of advanced sensor-based diagnostics, such as real-time electromyographic (EMG) monitoring, into routine mTBI assessments. The review highlights the advantages of utilizing multimodal data streams [33] in standard clinical evaluations to provide more objective and reliable measures of mTBI severity, potentially reducing the reliance on subjective assessments like symptom checklists and cognitive screening tests.

Additionally, the use of machine learning (ML) models to analyze complex neuromechanical data could help clinicians predict long-term outcomes for mTBI patients more accurately. Predictive analytics could identify patients at higher risk of prolonged recovery or persistent cognitive impairments, allowing for earlier interventions and more tailored rehabilitation plans [26]. For instance, a patient identified as having a high likelihood of chronic cognitive deficits based on early neuromechanical markers could be referred for intensive cognitive-behavioral therapy, reducing the long-term burden of mTBI.

An important implication in sports medicine is the adoption of more rigorous return-to-play criteria that account for neuromechanical recovery, rather than solely relying on self-reported symptoms or standardized cognitive assessments. Return-to-play protocols could be improved by incorporating objective metrics derived from neuromechanical models, such as RT recovery trajectories or motor coordination benchmarks [40]. The risk of premature return to play, which can increase the likelihood of sustaining subsequent concussions and long-term neurological damage, can thereby be mitigated. The insights gained from this review underscore the need for enhanced safety protocols. One practical recommendation is to implement real-time monitoring systems using wearable EMG, including the Pison READY, which enables intermittent neuromechanical evaluation during play [12]. Standalone RT testing devices like the Pison interface can conduct the necessary TBI testing on firmware (rather than via commercial testing software) to provide stable and accurate measurement of human reaction time and reduce the effect of device variability and software and stacking [12]. Such systems could flag early signs of mTBI, such as delayed reaction times or abnormal motor patterns, allowing for immediate intervention and preventing athletes from returning to play before they are fully recovered [41].

Rehabilitation programs for mTBI could benefit significantly from the use of personalized, data-driven approaches that leverage insights from neuromechanical models. This would allow clinicians to adjust therapy intensity and focus based on the patient’s progress in motor and cognitive recovery, as indicated by EMG or motion capture data [32]. The application of machine learning algorithms can further help identify the most effective interventions for unique patient subgroups. By analyzing neuromechanical patterns across large patient populations, these models could determine which rehabilitation exercises yield the best outcomes for patients with similar injury profiles, thereby personalizing rehabilitation protocols to optimize recovery [38].

In our systematic review, we found that due to the sensitive nature of research with pediatric subjects, the novelty of RT-based measures, and the experimental challenges in deconvoluting RT dynamics associated with variable motor–cognitive development stages and mTBI neurophysiology in pediatric populations, there were relatively few studies isolating pediatric populations in the broad literature survey conducted. We identified three studies that specifically examined reaction time (RT) as a diagnostic and prognostic metric in pediatric mild traumatic brain injury (mTBI). Brooks et al. (2016) found that slower RTs in the emergency department were predictive of persistent post-concussion symptoms at one month post-injury, suggesting that early RT assessments can inform recovery trajectories in youth [32]. Gazzellini et al. (2016) utilized time–frequency analyses of RT and EEG data, revealing significant low-frequency oscillations in RT among pediatric TBI patients, indicative of attentional lapses and potential disruptions in the default mode network [48]. Shultz et al. (2016) explored the relationship between processing speed, including RT, and adaptive functioning, highlighting that slower RTs were associated with deficits in executive function and daily living activities post-injury [69]. Collectively, these studies underscore the critical role of RT assessments in the pediatric mTBI population, emphasizing the need for age-appropriate, sensitive measures to detect subtle cognitive impairments and guide individualized rehabilitation strategies, but further robust experimental evidence is needed to make strong claims about the differences in mTBI neurophysiological drivers of RT characteristics between pediatric and adult populations.

Based on the review’s findings, several key recommendations emerge for the implementation of these practical changes. Clinicians should adopt a multimodal diagnostic approach, combining traditional clinical assessments with sensor-based neuromechanical tools to improve diagnostic accuracy and patient monitoring. In the sports context, athletic organizations should invest in wearable technology that monitors neuromechanical performance during and after competitions, integrating this data into return-to-play decisions to protect athletes’ long-term health.

Additionally, rehabilitation programs should incorporate continuous, real-time monitoring systems to track patient progress dynamically, allowing for more personalized and adaptive care. To support these advancements, healthcare systems and sports organizations should provide training and resources for clinicians and coaches to effectively utilize these technologies.

## 7. Conclusions

This systematic review and meta-analysis have provided a comprehensive evaluation of the neuromechanical probabilistic models used for characterizing mTBI, with a specific emphasis on RT as a diagnostic and prognostic metric. By assessing a diverse set of models—ranging from stochastic and generalized linear models to machine learning and time–frequency decomposition approaches—we have underscored the importance of quantitative, sensor-based assessments in understanding the complex interplay between cognitive and motor impairments post-mTBI. We seek to directly address unmet needs in neurotraumatology research such as those delineated by Dasic et al. [79], including support for early predictive assessment and the point-of-care collection and analysis of prognostic biomarkers, and increased longitudinal assessment post-mTBI, including telemedicine resources and individualized clinical pathways for patients with mild traumatic brain injury (mTBI) and its heterogeneity of natural history, presenting symptoms, and clinical trajectories can yield less granular field characterization. Our synthesis of neuromechanical modeling and sensor-based diagnostics highlights RT as a pathway toward efficient, low-cost personalized care with improved longitudinal diagnostic precision for tailored rehabilitation strategies. By bridging gaps in the assessment of cognitive–motor impairments and recovery, this work seeks to build clinical pathways in neurotrauma services and enhance the capacity of trauma centers to address mTBI [79].

Our findings indicate that RT is an effective non-invasive biomarker for mTBI, but existing models require refinement to account for the high sensitivity of modalities and variability in measurands. Inconsistencies remain in the types of physiological data used across studies, highlighting the need for standardized methodologies to enhance cross-study comparability. Further, integrating RT and other neuromechanical metrics into routine clinical practice could lead to more personalized diagnostic protocols, reducing reliance on subjective symptom assessments and providing objective indicators of injury severity and recovery trajectories. Additionally, these models offer the potential for refining return-to-play criteria in sports and improving patient outcomes through more tailored rehabilitation programs that leverage real-time neuromechanical monitoring.

The review also identified critical gaps in the literature concerning long-term outcomes following repetitive mTBI and the integration of predictive modeling into large-scale clinical settings. These include existing challenges with RT measurements, which, through enhanced tools, including EMG, can be made simpler, more cost-effective, and easier to administer. These can enable the capturing of chronic effects through advanced computational frameworks capable of handling high-dimensional and dynamic neuromechanical data. Further, efficient measurement modalities can promote wide-scale continual baseline RT assessments, such as during general physical medical examinations, so that the sensitivity of RT to cognitive–motor slowing in and after repetitive mTBI can be mitigated [80,81]. Such a well-validated and robust RT test can foster collaborative efforts between clinicians, engineers, and data scientists and lead to the development of models that are scalable and clinically translatable, ensuring that mTBI diagnostics can effectively address individual variability.

While significant progress has been made in characterizing mTBI through neuromechanical models, this review emphasizes the need for more rigorous, standardized approaches that can bridge the gap between research and clinical practice. By refining existing models and integrating new technologies, more accurate, efficient, and patient-centered approaches to mTBI diagnosis, treatment, and recovery can be identified.

Our findings underscore that, while TFD models currently hold the highest promise for clinical application in mTBI assessments, an integrated multi-model approach—leveraging both TFD and stochastic models—could yield the most comprehensive insights. For researchers, this means prioritizing robust signal preprocessing, particularly noise reduction techniques, and refining experimental protocols to control for confounding factors, such as variability in baseline cognitive and motor function across subjects. The standardization of task design in RT assessments is also crucial, as inconsistent stimulus complexity and response requirements can significantly impact model performance.

Moving forward, researchers should aim to build larger datasets and consider combining TFD with stochastic frameworks that allow for real-time adaptability and generalizability across diverse populations. Clinical studies can benefit from implementing these hybrid models as part of routine mTBI assessments, while athletic and rehabilitation programs may utilize TFD models to track longitudinal recovery and optimize return-to-play protocols. Ultimately, this review provides a clear recommendation: to advance mTBI diagnostics and treatment, the field must embrace models that can manage high-dimensional, time-variant data, with TFD and stochastic methods leading the way toward more accurate, cost-effective mTBI assessments.

## Figures and Tables

**Table 1:** Summary of abbreviations used in this work.

Acronym	Definition
AUC	Area Under the Receiver Operating Characteristic Curve
CT	Computed Tomography
DAI	Diffuse Axonal Injury
DTI	Diffusion Tensor Imaging
EMG	Electromyography
fMRI	Functional Magnetic Resonance Imaging
GCC	General Cognitive Constraints
GCS	Glasgow Coma Scale
GLM	Generalized Linear Model
LGR	Logistic Regression
LNR	Linear Regression
MCH	Morphological Change
ML	Machine Learning
mTBI	Mild Traumatic Brain Injury
MTP	Motor Task Performance
NAM	Neural Activity Metrics
NOS	Newcastle-Ottawa Scale
PCS	Post-Concussion Syndrome
RoB	Risk of Bias
ROC	Receiver Operating Characteristic
RTC	Reaction Time Constraints
RT	Reaction Time
SBC	Stimuli-Based Constraints
SCAT	Sport Concussion Assessment Tool
SVM	Support Vector Machine
TFD	time–frequency Distribution

**Table 2:** Bias assessment of selected studies.

Study:	D1	D2	D3	D4	S	R	E	C	A	F
Rier et al. [22]										
Cui et al. [23]										
Dean et al. [24]										
Dumphy et al. [25]										
Yu et al. [26]										
Halterman et al. [27]										
Caccese et al. [28]										
Gould et al. [29]										
Feller et al. [30]										
Pontifex et al. [31]										
Brooks et al. [32]										
Maruta et al. [33]										
Kontos et al. [34]										
Arciniega et al. [35]										
Arrieux et al. [36]										
Kelly et al. [37]										
Mayer et al. [38]										
Cui et al. [39]										
Frencham et al. [40]										
Adams et al. [41]										
Shen et al. [42]										
Bartsch et al. [43]										
Rogers et al. [44]										
Truong et al. [45]										
DeWitt et al. [19]										
Caccese et al. [46]										
Henry et al. [47]										
Gazzellini et al. [48]										
Dean and Sterr [49]										
Brooks et al. [50]										
Pearce et al. [51]										
Mayer et al. [52]										
Stokum et al. [53]										
Vanderploeg et al. [54]										
Fischer et al. [55]										
Tsirka et al. [56]										
Churchill et al. [57]										
Lempke et al. [58]										
Favorov et al. [59]										
Norman et al. [60]										
Kaukas et al. [61]										
Warlick et al. [62]										
Balaban et al. [63]										
Piponnier et al. [64]										
Tommerdahl et al. [65]										
Sosnoff et al. [66]										
Reches et al. [67]										
Kerwin et al. [68]										
Shultz et al. [69]										
Mcintire et al. [70]										
Nelson et al. [71]										
Cole et al. [72]										
DeHaan et al. [73]										
Swenson et al. [74]										
Womack et al. [75]										
Drew et al. [76]										
Manelis et al. [77]										

Bias assessment of selected studies(cont.d): gray indicates high risk of bias, white cells indicate low risk.

**Table 3:** Meta-analysis results by model type, input, and output.

Model	Sample Size (Range)	*p*-Value (Range)	Stouffer Z-Score	BMA Weight (Avg.)
STM	5–3214	0.0001–0.5	2.35±0.45	0.75±0.12
LNR	5–451	0.001–0.0245	2.54±0.58	0.80±0.21
ML	8–18	0.04–0.05	2.01±0.32	0.65±0.16
TFD	5–3214	0.0001–0.0438	2.10±0.35	0.70±0.18

**Table 4:** Summary of neuromechanical probabilistic models with time per data point (ranges).

Model	Time Complexity	Memory Usage	Time Per Data Point (s)	Scalability
STM	O(n·*p*·k)	O(n·p)	$10^{-5}$–$10^{-3}$	High
GLM	O(n·*p*·k)	O(n·p)	$10^{-5}$–$10^{-3}$	High
ML	O(L·*n*·p)	O(L·*n*·p)	$10^{-4}$–$10^{-2}$	High
LNR	$O(n^{3})$	$O(n^{2})$	$10^{-3}$–$10^{-2}$	Low
TFD	O(nlogn)	O(n)	$10^{-5}$–$10^{-3}$	Moderate
ANV	O(n)	O(n)	$10^{-6}$–$10^{-4}$	Moderate

**Table 5:** Summary of signal characteristics for inputs and outputs.

Signal	Key Characteristics	Amplitude/Range	Resolution/Frequency
NEU	High spatial resolution, dynamic range	FA: 0–1	1–2 mm voxel, 1–2 s TR
MOT	High sampling rate, low latency	−16 g to +16 g	100–1000 Hz
COG	Task-dependent accuracy and timing	Variable	Millisecond precision
CPM	Accuracy, error rates	N/A	Millisecond precision
NAM	Sub-millisecond to second-level timing	Variable	Sub-millisecond (EEG), 1–2 s (fMRI)
MTP	Movement amplitude, velocity, acceleration	Variable	Sub-millisecond precision

## Data Availability

Data sharing is not applicable to this article as no new data were created or analyzed in this study.
